# A Comparison of Hemoglobin A_2_ Levels in Untreated and Treated Groups of HIV Patients on ART Including Zidovudine

**DOI:** 10.1155/2013/828214

**Published:** 2013-12-26

**Authors:** Jitendra Singh Nigam, Jyotsna Naresh Bharti, Dinker Kumar, Ankit Sharma

**Affiliations:** ^1^Department of Pathology, D.D.U. Hospital, Hari Nagar, New Delhi 110066, India; ^2^Department of Pathology, Maulana Azad Medical College, New Delhi 110002, India

## Abstract

*Objective*. To assess the level of haemoglobin A_2_ in HIV patients on antiretroviral therapy (ART) including zidovudine with untreated HIV patients. *Material and Methods*. The study was a case control study. A total of 185 patients were included in the study; the case group included 125 HIV patients who were on antiretroviral therapy (ART) including zidovudine and 60 were in the control group who were not receiving ART. The high-performance liquid chromatography (HPLC) was done and hemoglobin A_2_ level was observed; value more than 3.5% was considered significant. The Hb A_2_ percentages of HIV patients were compared with those of control using an unpaired *t*-test. Results. The mean of Hb A_2_ in case group was 3.462% (SD 0.675) and in control group it was 2.815% (SD0.246). The higher Hb A_2_ value was seen in significant number of treated patients than control group (*P* < 0.0001). Conclusion. The clinicians, pathologists, haematologists, and genetic counsellors should be aware of effects of nutritional anaemia and ART on Hb A_2_ to reduce the chances of misdiagnosis of **β**-thalassaemia especially in developing countries and for centres for antenatal screening.

## 1. Introduction

Cooley and Lee were the first to describe the diminished rate of synthesis of one or more globin chains or its part, which consequently results in reduced rate of synthesis of the haemoglobin. Whipple and Bradford in 1936 gave the term “Thalassaemia” [1]. *β*-thalassaemia is commonly seen in Mediterranean region, Indian subcontinent, Southeast Asia, and African ancestry [1]. For *β*-thalassaemia, screening of populations was done either by measuring the haemoglobin A_2_ percentage or by red cell indices in patients with MCV or MCH below a certain cut-off point [1]. The haemoglobin A_2_ is 4%-5% in most cases of heterozygous *β* ^0^ or severe *β* ^+^ thalassaemia and 3.6–4.2% in heterozygous mild *β* ^+^ thalassaemia [1]. The raised Hb A_2_ values are seen in unstable haemoglobins, hyperthyroidism, and megaloblastic anaemia, and human-immunodeficiency-virus- (HIV-) infected patients on antiretroviral therapy, while hypochromic microcytic red cell picture is seen only in heterozygous *β*-thalassaemia [2]. History, clinical details, and coexisting condition help to rule out the causes of increase in Hb A_2_, which can avoid unnecessary investigation and reduce the financial burden of institutional setting in developing countries. Awareness of the fact that there are increases in Hb A_2_ due to antiretroviral drugs among clinicians and pathologists should be must. Spiga et al. reported that zidovudine inhibits *β*-globin gene expression in human erythroid progenitor cells [3]. HIV-infected patients receiving highly active ARV therapy (HAART) or ZDV are associated with elevation of Hb A_2_ value [2–5]. Galacteros et al. observed that HIV-infected patients at stage IV of disease have moderate elevation of Hb A_2_ level and beta-thalassemia-like unbalanced biosynthetic globin ratio [6].

## 2. Materials and Methods

This study was a case control study. A total of 185 patients were included in the study, who were recruited from the ART center of the DDU Hospital. The case group included 125 HIV patients who were on antiretroviral therapy (ART) including zidovudine, nevirapine, and lamivudine for the last three years and 60 untreated newly diagnosed patients were in the control group who were not receiving ART. All these patients were tested with rapid ELISA method for HIV and V.D.R.L for syphilis. In case group, 75 patients were male and 50 were female. The complete clinical data, haemogram, osmotic fragility (OF) test, and biochemical investigations were done. All the patients were tested for HIV. The red cell indices, red cell distribution width (RDW), and OF test were done for the evaluation of thalassaemia. High-performance liquid chromatography (HPLC) was done and hemoglobin A_2_ level was observed; value more than 3.5% was considered significant. The Hb A_2_ percentages of HIV patients were compared with those of control using an unpaired *t*-test. HbE trait, history of alcohol, and abnormal liver function test were the exclusion criteria for the study.

## 3. Results

The red cells indices show mean MCV 96.88 fl, and 54.4% of (68/125) patients had MCV > 100 fl. The rest of the patients had MCV within normal range. In control group, all patients have MCV within normal range with mean value of MCV 82 fl. RDW is increased in 60.8% (76/125) of patients with mean RDW 20.9. Serum ferritin, folic acid levels, and vitamin B12 level were within normal limits. The mean of Hb A_2_ in HIV patients on treatment was 3.462% (SD 0.675) and control has a mean Hb A_2_ of 2.815% (SD 0.246). The high Hb A_2_ was seen in significantly higher number of patients than controls (*P* < 0.0001). Osmotic fragility (OF) test of all patients and control was normal (Table 1).

## 4. Discussion

The *β*-thalassemia is one of the most common single gene disorders in India with an overall prevalence of 3-4%, and *β*-thalassemia carriers vary between 8 and 10% in certain communities like Sindhis, Muslims, Cutchi Bhanushalis, and some tribal groups [7]. The diagnosis of heterozygous *β*-thalassaemia is of key importance in the antenatal setting because it is helpful in concern to haemoglobinopathy counseling, partner screening, and possibly extensive haematological and molecular analyses of the patient samples [2]. Rao et al. observed that patients with megaloblastic anaemia have raised Hb A_2_  (*P* < 0.001) as compared to normal cases [8]. In the present study, vitamin B_12_ and folic acid levels were within normal limits in both study and control groups, but mean MCV in study group was 96.88 fl and 54.4% showed macrocytosis (MCV > 100 fl). The impair DNA synthesis in megaloblastic anaemia occurs due to delayed nuclear maturation, while zidovudine causes inhibition of nucleoside reverse transcriptase [9]; therefore, in both conditions, the increased synthesis of *δ* chains leads to higher Hb A_2_ values because more Hb synthesis occurs in less mature erythroid precursors, and the synthesis of *δ* chains is relatively greater in less mature cells [10]. Thyroid hormone affects the *δ* gene transcription and causes increase in both the percentage and absolute amount of Hb A_2_ [9]. In the present study, thyroid functions test was within normal limits in both study and control groups. Wilkinson et al. observed that incidence of increased Hb A_2_ value was significantly high in patients treated with zidovudine containing HAART than in those treated with non-zidovudine HAART and normal volunteers [4]. In the present study all patients on zidovudine containing ART had raised Hb A_2_ value than control group. 37.33% (28/75) of the males and 24% (12/50) of the females showed raised Hb A_2_. Pornprasert et al. also observed that ART increases the Hb A_2_ value and alters some haematological parameters that might affect the diagnosis of thalassaemia carriers, especially in the investigation of *β*-thalassaemia trait [11]. Kosalaraksa et al. observed that abnormal high % Hb A_2_was found in more than a half of ZDV-exposed HIV-infected children; therefore, low MCV and MCH were important coparameters to reduce the misinterpretation of *β*-thalassemia trait; however, the DNA analysis should be performed to confirm the diagnosis in such situation [12]. Howard et al. concluded that Hb A_2_ should be estimated before the initiation antiretroviral drugs in all HIV-infected women of child-bearing age to allow a diagnosis of heterozygous *β*-thalassaemia to be made and help to prevent the inaccurate diagnosis of heterozygous *β*-thalassaemia, and unnecessary genetic counselling and genetic analysis [2]. Pornprasert et al. also concluded that Hb A_2_ values should be measured in all HIV-1-infected couples before the initiation of antiretroviral drugs to rule out misdiagnosis of *β*-thalassaemia [13]. In the present study, all cases showed normal Hb A_2_ before starting ART. Few drugs have effect on various haemoglobins like butyrate, which induces fetal hemoglobin (HbF) synthesis in cultures of erythroid progenitors in man by beta oxidation by mitochondrial enzymes resulting in the formation of two acetate molecules from each molecule of butyrate [14]. The fatty acid analogue valproic acid (*n*-dipropylacetic acid) may increase the synthesis of fetal hemoglobin [15]. Macrocytosis was a striking result of hydroxy urea therapy and paralleled the increase in HbF synthesis [16]. In the present study, no history of intake of other drugs was present.

## 5. Conclusion

In India, HIV patients have wider access to ART to reduce the annual AIDS-related deaths, and all patients with increased Hb A_2_ should be investigated for detailed history, identified high risk factor for HIV, and all haematological parameters including OF test, MCV, MCH, MCHC, and RDW. The clinicians, pathologists, haematologists, and genetic counsellors should be aware of effects of nutritional anaemia and ART on Hb A_2_ to reduce the chances of misdiagnosis of *β*-thalassaemia.

## Figures and Tables

**Table 1:** Results.

	Case	Control
Number of patients	125 (75 M/50 F)	60
Mean MCV	96.88 fl	82 fl
MCV > 100 fl	54.4% (68/125)	Nil
Mean RDW	20.9	14.6
RDW ↑	60.8% (76/125)	16.67% (10/60)
Serum ferritin	Normal	Normal
Folic acid	Normal	Normal
Vitamin B_12_	Normal	Normal
Mean Hb A_2_	3.462% (SD 0.675)	2.815% (SD 0.246)
<3 Hb A_2_	11.2% (14/125)	70% (42/60)
3–3.5 Hb A_2_	56.8% (71/125)	30% (18/60)
>3.5 Hb A_2_	32% (40/125) 37.33% (28/75) male; 24% (12/50) female	None
Osmotic fragility (OF)	Normal	Normal
